# Spontaneous object-location memory based on environmental geometry is impaired by both hippocampal and dorsolateral striatal lesions

**DOI:** 10.1177/2398212820972599

**Published:** 2020-11-17

**Authors:** Steven L. Poulter, Yutaka Kosaki, David J. Sanderson, Anthony McGregor

**Affiliations:** 1 Department of Psychology, Durham University, Durham, UK; 2 Department of Psychology, Waseda University, Tokyo, Japan

**Keywords:** Spontaneous object recognition, context, geometry, cognitive map, hippocampus, dorsolateral striatum

## Abstract

We examined the role of the hippocampus and the dorsolateral striatum in the representation of environmental geometry using a spontaneous object recognition procedure. Rats were placed in a kite-shaped arena and allowed to explore two distinctive objects in each of the right-angled corners. In a different room, rats were then placed into a rectangular arena with two identical copies of one of the two objects from the exploration phase, one in each of the two adjacent right-angled corners that were separated by a long wall. Time spent exploring these two objects was recorded as a measure of recognition memory. Since both objects were in different locations with respect to the room (different between exploration and test phases) and the global geometry (also different between exploration and test phases), differential exploration of the objects must be a result of initial habituation to the object relative to its local geometric context. The results indicated an impairment in processing the local geometric features of the environment for both hippocampus and dorsolateral striatum lesioned rats compared with sham-operated controls, though a control experiment showed these rats were unimpaired in a standard object recognition task. The dorsolateral striatum has previously been implicated in egocentric route-learning, but the results indicate an unexpected role for the dorsolateral striatum in processing the spatial layout of the environment. The results provide the first evidence that lesions to the hippocampus and dorsolateral striatum impair spontaneous encoding of local environmental geometric features.

## Introduction

The hippocampus (HPC) is often said to support a cognitive map of the environment (O’Keefe and Nadel, 1978; Poulter et al., 2018) but what exactly is meant by a cognitive map is more equivocal (Bennett, 1996; Mackintosh, 2002). If a cognitive map is a representation of the inter-relations among stimuli in the environment (Leonard and McNaughton, 1990), an animal that possessed one could represent the global layout of the environment. This notion is captured in the interpretation that when animals navigate relative to environmental geometry, they do so based on a configural representation of the shape of the environment, abstracted from the elements creating it (Cheng and Spetch, 1998; Gallistel, 1990).

To test whether animals form a representation of macroscopic geometric relations, animals trained in one environment are tested in another, which shares some of its geometric features with that of the training environment. Crucially, the test environment differs in its global shape to the training environment, so non-random search at test indicates that animals did not rely solely on a global representation of space in the initial exposure to the training environment, but instead were guided by local spatial features that the two environments shared (McGregor et al., 2006; Pearce et al., 2004; Tommasi and Polli, 2004). Pearce et al. (2004) and subsequently Jones et al. (2007) demonstrated that lesions to the HPC impaired navigation based on these local geometric properties of space.

However, there is evidence of both global and local geometry controlling spatial behaviour in pigeons (Bingman et al., 2006), chicks (Kelly et al., 2011) and humans (Lew et al., 2014; Sturz et al., 2012). Sotelo et al. (2019), based on Pearce et al. (2004), trained pigeons to locate a reinforcer in one corner of a rectangular arena before transferring them to a kite shape, in which two corners shared the same local geometric properties as the corners in the rectangle. Unlike Pearce et al. (2004), who found transfer of rats’ search between environments, there was no evidence that pigeons recognised the matching local geometry between the two arenas, despite learning the correct location within the rectangle. Furthermore, immediate early gene (IEG) analysis of hippocampal c-Fos activation was higher for pigeons exposed to the familiar rectangle compared with others exposed to an unfamiliar trapezoid, suggesting that they recognised the overall shape of the rectangle but not of the trapezoid, and that this recognition was associated with hippocampal activity.

Therefore, the role of the HPC in learning based on local or global geometry remains uncertain. One difference between Pearce et al. (2004) and Sotelo et al. (2019), other than the species tested, was the use of appetitive and aversive procedures, which could have altered the way the animals behaved (Golob and Taube, 2002). To remove the confound of motivational reinforcer, we employed a spontaneous object recognition (SOR) version of a geometry learning task (Experiment 1). We tested three groups – one with lesions to the HPC, a sham-operated control group, and one with lesions to the dorsolateral striatum (DLS). The DLS group was included because there is little information on the effect of DLS lesions on object recognition. Korol et al. (2019) recently examined the role of the DLS in SOR and found no evidence for its involvement in object-location memory, so inclusion of this group acts as a positive control. However, Kosaki et al. (2015) found that DLS lesions significantly facilitated place learning in a swimming pool, so one possibility was that we would see a similar facilitation in the current study.

## Experiment 1

### Method

#### Subjects

The subjects were 32 male Lister hooded rats (*Rattus norvegicus*) supplied by Charles River (UK). They were approximately 3 months of age when surgery was performed, and 5 months old when testing on the current experiment was carried out. They had been previously used in an unrelated water maze task. The rats were housed in pairs in a light-proof, temperature-controlled room (20°C), with the lights turned on at 07:00 h and off at 21:00 h. Testing was conducted when the lights were turned on in the home room. All animals were provided with ad libitum access to food and water. In total, 12 rats received lesions to the HPC, 12 received lesions to the DLS, and 8 were sham-operated controls. The experiment was conducted in accordance with the Animals (Scientific Procedures) Act 1986 and Home Office and institutional guidelines.

#### Surgical procedure

Each rat was deeply anaesthetised with a mixture of isoflurane (5%) and oxygen (2 L/min), and its scalp was shaved. It was then secured into a stereotaxic frame (Kopf Instruments, Tujunga, CA, USA), with the incisor bar set at −3.3 mm. The anaesthetic was reduced to a maintenance concentration (1%–2% isoflurane at 0.8 L/min), and the animal’s heart rate and reflexes were closely monitored throughout to make certain the rat remained at the appropriate level of anaesthesia. An incision was made along the midline of the scalp and the bone covering the neocortex was removed using a dental burr. An arm was mounted on to the stereotaxic frame to which was attached a 2-µL Hamilton syringe attached to an electronic microdrive (model KDS 310; KD Scientific, New Hope, PA). The microdrive controlled the quantity (0.05–0.25 µL) and rate (0.03 µL/min) of excitotoxin. Ibotenic acid (Tocris Bioscience, Bristol, UK), dissolved in phosphate-buffered saline (pH 7.4) to produce a 63 mM solution was infused in 28 and 12 injection sites for each bilateral hippocampal and dorsolateral lesion, respectively. The infusion coordinates for the hippocampal lesions are reported in Coutureau et al. (1999) and the DLS lesions in Kosaki et al. (2015). The needle was left in place for 2 min following each infusion to permit diffusion of the ibotenic acid into surrounding tissue. Sham-operated controls underwent similar surgical procedures as for the HPC and DLS rats, with incision of the skin, neocortex exposed, and dura perforated using a needle, but no infusions were made. The incision was sutured at the end of the procedure and the rat was placed into a warm chamber to recover. Each rat was administered subcuticular Buprenorphine (0.01 mg/kg) pre- and post-procedure to provide analgesia, and a post-procedure subcuticular 10-mL saline and glucose solution to aid rehydration. Once sufficiently recovered, the rat was transferred back to its home cage. A minimum of 14 days postoperative recovery was allowed before behavioural testing began.

At the end of the experiment, rats were deeply anaesthetised with sodium pentobarbitone (200 mg/kg) and perfused transcardially with 0.9% saline followed by 4% paraformaldehyde solution (0.1 M phosphate-buffered). Brains were removed and stored in 4% paraformaldehyde solution (0.1 M phosphate-buffered) for several days before being transferred to 25% sucrose (in 0.1 M phosphate-buffered saline) for 24–48 h before being sectioned (40 µm), mounted on slides, and stained with cresyl violet.

#### Apparatus

The apparatus was identical to that reported by Poulter et al. (2013). Briefly, a kite-shaped arena occupied one testing room and a rectangular arena occupied another. The testing rooms had similar dimensions (approximately 290 × 185 × 260 cm^3^ high) and each had a speaker mounted on the wall to provide white noise, together with a table in the corner on which rats were held. Each room was lit by a lamp that was placed on the floor with an 11 W bulb and was positioned such that shadows were not cast into the arena. A camera was attached to a rail above each arena, and images were transmitted to a monitor and recorder that were located in an adjacent room. The arenas were made from medium-density fibreboard and were painted light grey. Each arena was made up of two long walls (100 × 50 cm^2^ high) and two short walls (50 × 50 cm^2^ high). The walls in the kite were arranged such that the corners where the long and short corners met were at an angle of 90°, so that they were geometrically identical to the corners in the rectangle (see Figure 2). The arenas were located on the floors of the testing rooms and could be rotated to occupy four different positions along a north-south or east-west axis.

Junk objects, including bottles, metal clips, ceramic ornaments and small toys, occupied the corners of the arenas. Objects were chosen to be similar in terms of materials and dimensions within a trial. They were affixed to the arena floor using Velcro. Multiple versions of the same objects were created so that different versions of the same object were presented in different arenas during the sample and test phases.

#### Procedure

Rats were transported into the test laboratory, four at a time, in a holding cage comprising a Perspex bottom and wire top. While transporting animals to and from the testing rooms, a fleece cover was placed over the cage to minimise the stress caused by this movement. Throughout behavioural procedures, the holding cage and rats, when not being tested, were placed on a table in the corner of the room. Each trial commenced with the experimenter, always approaching the arena from the same southerly direction, placing the rat gently into the centre of the arena. After the trial commenced, the experimenter left the testing room and waited in an adjacent room until the trial ended. On completion of the trial, the animal was removed from the arena and placed back into the holding cage.

Rats received five sessions of habituation prior to beginning the experimental stage of the experiment. The first session of habituation consisted of pairs of animals being placed into the rectangular arena, then into the kite, for 5 min in each. Sessions two to five of habituation followed the same procedure as Session 1 with the exception that animals were now allowed to explore each arena individually. Between each session of habituation, each arena was rotated 90° anticlockwise to ensure all rats explored the empty arenas in each of the four possible orientations. Each session of habituation took place on a separate day, and animals were run in the same order throughout. The arenas were wiped down with dry paper towelling prior to each animal or pair of animals beginning exploration. At the end of each testing day, both arenas were cleaned with alcohol wipes.

Following habituation, the experimental stage began, in which animals received one object recognition trial per day for 4 days. In the sample phase, each rat was exposed to two different objects, A and B, in corners E and G of the kite, for 2 min (see Figure 2). After a squad of four rats had completed the sample phase, they were then transported to the adjacent testing room for the test phase. In the test phase, which lasted for a further 2 min, each rat was placed in the rectangle arena in which two identical copies of one of the objects were presented in the right-angled corners J and K. The retention interval between the sample and test phase for each rat was approximately 8 min. The orientation of the rectangle changed between days but remained constant for all animals on the same day. Only two of the four possible kite orientations were used on any given day, although it was ensured that each orientation was counterbalanced equally between all animals. For the test phase, animals were split into equal groups so that half received object A at test and the remainder object B, and, in so doing, ensured that the novel location (corner J or K of the rectangle) was also assigned equally between animals. Thus, for each individual rat, the novel object-location corner changed daily. Therefore, any preference for exploration of one object over another could not be explained by the positions of the objects with respect to generalisation between extramaze cues or by a preference for one right-angled corner over another. On completion of a trial by an animal and prior to the next animal beginning their trial, each object was thoroughly cleaned with alcohol wipes and the arena was wiped down with dry paper towelling. At the end of each testing day, both arenas were cleaned with alcohol wipes.

Ethovision (version 3.1) software was used to track the movement of each animal in the test phase. For each 120-s test phase, the time a rat spent within a circular zone centred on each of the objects was recorded. There was a gap of approximately 5 cm between the edge of the object and the perimeter boundary of the zone. Thus, the time an animal spent within an area of 5 cm from the object was recorded. To ensure tracking indexed exploration only, time spent in the zone was only recorded if the rat’s head entered either of these circular zones. This was automated by the Ethovision software. It was also possible for the software to record when the rat was not actively exploring, but instead engaged in other activities, such as grooming. However, the tracking system stopped tracking when contrast was lost between the arena floor and the rat’s head, which occurred if the rat was rearing. While we are confident that the automated tracking captured active exploration, it may be that what we term ‘exploration’ may include other behaviours.

#### Statistical analysis

The time rats spent in the vicinity of each object was recorded for each of the four 120-s test phases. Mean time spent in the vicinity of each object over the four test phases is reported. There was no minimum object exploration criterion applied for each trial, but mean individual exploration across days varied between 15.3% and 33.1% of the test phase duration. All data were analysed using two-way analysis of variance (ANOVA). For Experiment 1, we predicted a group × object interaction. Interactions were analysed using simple main effects analysis using the pooled error term from the original ANOVA.

### Results

**Figure 1.**
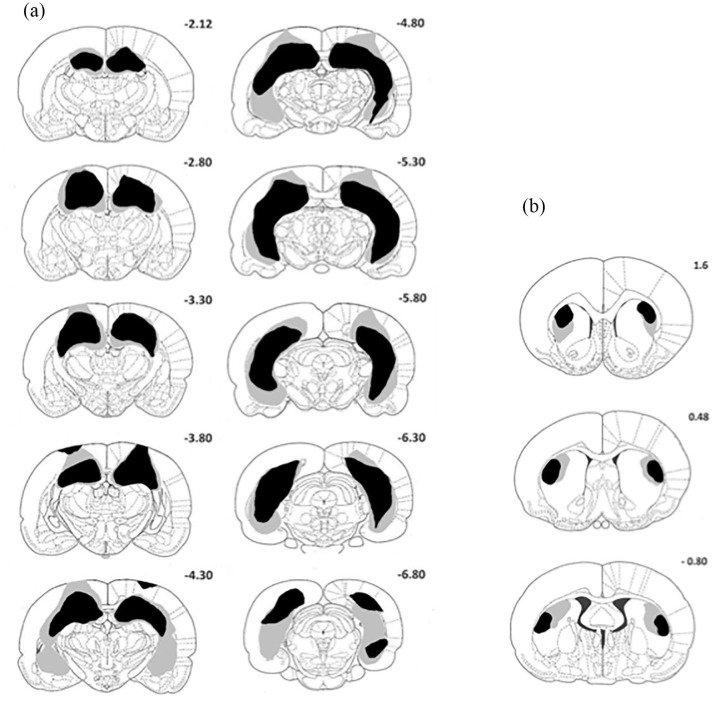
Coronal sections displaying the extent of (a) hippocampal damage and (b) DLS damage. The case with the largest (grey shading) and smallest (black shading) amount of tissue loss is represented for each lesion group. The numbers refer to the distance anterior or posterior to bregma for each section, according to Paxinos and Watson (2007).

Figure 1 depicts reconstructions of the minimum (black shading) and maximum (grey shading) extent of hippocampal (A: left-hand panel) and DLS (B: right-hand panel) lesions on a series of coronal sections. Rats in group HPC all sustained bilateral damage to the dorsal and ventral HPC (CA fields 1–4), the dentate gyrus and the subicular cortices. The main sparing of hippocampal tissue was observed in the most medial areas of the dorsal HPC. One rat received lateral damage in both hemispheres that extended into the lateral entorhinal and perirhinal cortices, so this animal was excluded from the analysis. In the majority of the remaining 11 rats, there was damage to the cortical area overlying the dorsal HPC. This typically included partial damage to motor, visual, somatosensory, parietal and retrosplenial agranular cortices (for reports of similar extrahippocampal damage in hippocamptomised rats, see Albasser et al., 2012; Iordanova et al., 2009). Similar to Albasser et al. (2012), the partial cortical damage described above left plenty of sparing in each of these areas. For rats in group DLS, visible widening of the lateral ventricles was observed in all cases owing to tissue shrinkage caused by the lesion. Inspection of the stained tissue revealed that the intended lesion site was off target in three rats. In these cases, which were excluded from subsequent analysis, there was significant extrastriatal damage to cortical areas adjacent to the DLS. In the remaining rats, cell loss and modest gliosis was found in the targeted area. Thus, there were 11, 9 and 8 rats included in the behavioural analyses for group HPC, DLS and Sham, respectively.

**Figure 2.**
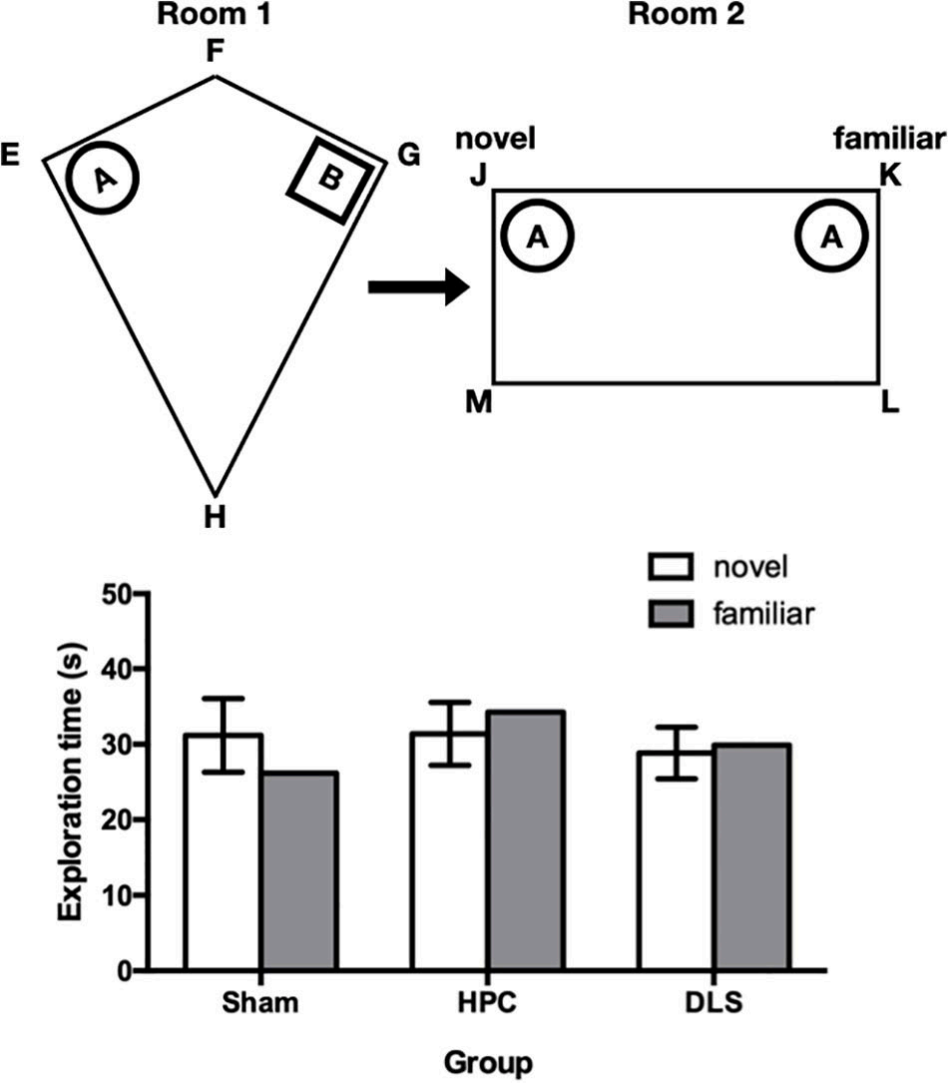
The upper panel shows a schematic diagram showing the design of Experiment 1. The arenas were in different experimental rooms. Objects A and B are represented by circular and square symbols, respectively. Preferential exploration of object A in corner J of the rectangle over the identical object in corner K indicates the animal’s detection of its novel location, despite the fact both of the objects were placed in a differently shaped arena in a different room. The lower panel shows the mean exploration times of each of the two test objects for each of the three groups. The error bars show the 95% confidence interval for the mean within-group difference between exploration times for the two objects, based on the pooled error term.

To simplify the account of the behavioural results, it will be assumed, as shown in the upper panel of Figure 2, that rats were exposed to objects A and B in corners E and G of the kite, before being presented with two copies of object A in corners J and K of the rectangle. In fact, however, the locations of objects A and B in the sample phase and the identity of the object (A or B) at test were counterbalanced. With reference to Figure 2, corner E of the kite is the geometric equivalent of corner K in the rectangle because in both corners, the long wall is to the left of a short wall. Thus, it was expected that object A in corner J of the rectangle would be explored more than object A in corner K, as it was in a novel location relative to the local geometric cues provided by the arena, whereas object A in corner J was in a familiar location.

The mean times that rats in each group spent in the vicinity of objects in the novel and familiar locations in the test phase are shown in the lower panel of Figure 2. Overall, exploration was similar between groups, but the Sham group appeared to spend more time in the vicinity of the object in the novel location compared with the familiar location. Neither the HPC or DLS groups appeared to discriminate locations. A two-way ANOVA of mean object exploration times over the four test trials, with group (Sham, HPC and DLS) and object location (novel, familiar) as factors, failed to reveal a significant group × object location interaction, F(2, 25) = 3.36, p = 0.051, but as there was an a priori prediction that the groups would differ, planned comparisons were used to examine group differences. Analysis of simple main effects using the pooled error term revealed that group Sham spent more time near the object in the novel than the familiar location, F(1, 25) = 4.44, p = 0.045, but that groups HPC, F(1, 25) = 2.06, p = 0.16, and DLS, F(1, 25) = 0.21, p = 0.65, did not. These differences are illustrated more clearly in the lower panel of Figure 2, which shows group means for time spent near the object in the novel and familiar location, along with the 95% confidence interval for the mean difference between the exploration times, shown arbitrarily on the novel object mean bar. The main effects of object location, F(1, 25) = 0.08, p = 0.79, and group, F(2, 25) = 1.83, p = 0.18, were not significant.

The results indicate an impairment for both HPC and DLS groups, compared with group Sham. Before discussing the results further, we report Experiment 2, designed as a control to ensure that the lesions did not impair discrimination based on a non-spatial version of the procedure.

## Experiment 2

### Method

**Figure 3.**
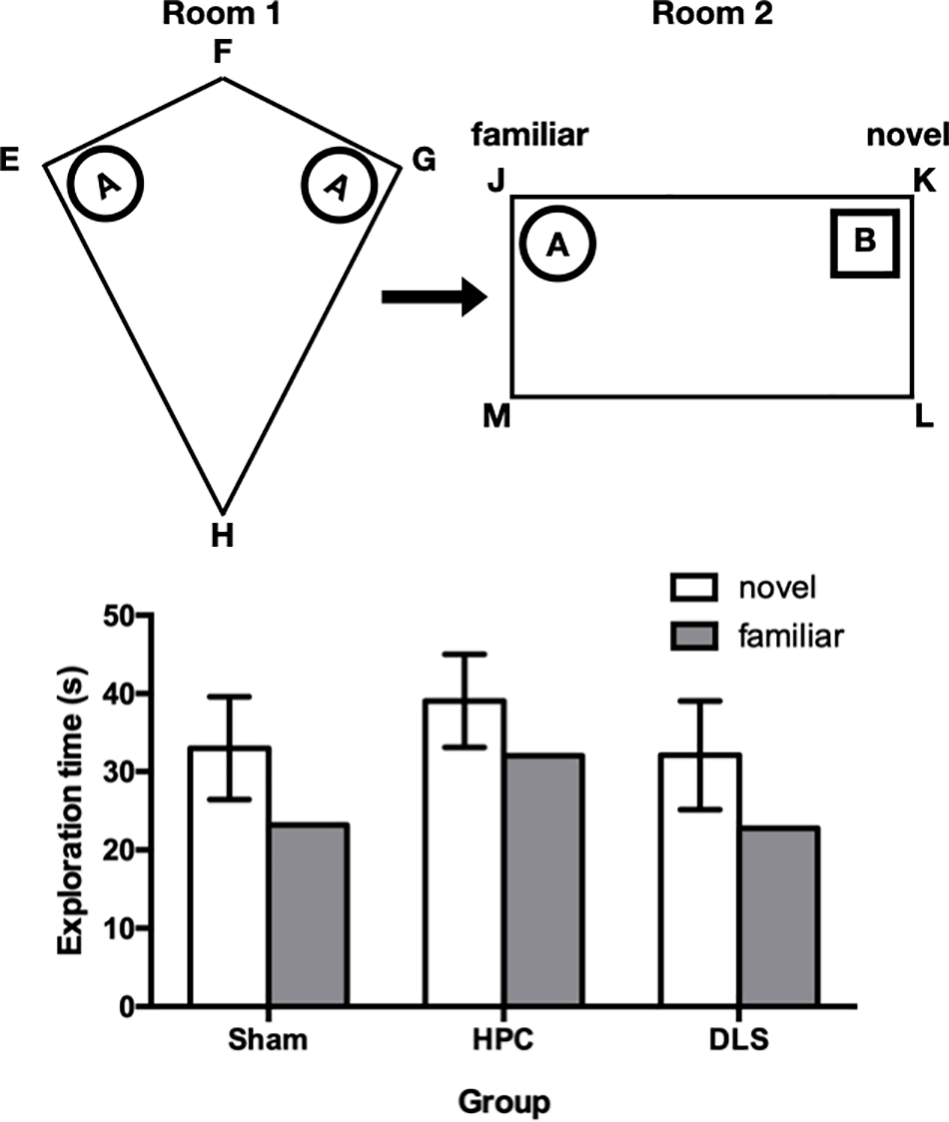
The upper panel shows a schematic diagram showing the design of Experiment 2. The two arenas were housed in different rooms. Objects A and B are represented by circular and square symbols, respectively. Preferential exploration of object B over object A indicates the animal’s detection of its novelty. The positions of objects A and B varied over trials and between rats so the positions of the objects relative to the local geometric features of the rectangle were irrelevant for successful performance. The lower panel shows the mean exploration times of each of the two test objects for each of the three groups. The error bars show the 95% confidence interval for the mean within-group difference between exploration times for the two objects, based on the pooled error term.

The subjects and apparatus were identical to Experiment 1. The procedure was identical to Experiment 1 with the following exceptions. First, instead of undergoing the same habituation phase as in Experiment 1, rats were given a single refresher habituation session, which involved them spending 5 min in their holding case in each testing room. Second, for the experimental stage, instead of being presented with two different objects in the right-angled corners of the kite, in Experiment 2, rats were presented with two copies of object A in corners E and G of the kite, followed by objects A and B in corners J and K of the rectangle. In essence, this procedure emulates a standard object recognition procedure, but with the equivalent changes in global and local context that were encountered by rats in Experiment 1. A schematic representation of the design of Experiment 2 is shown in the upper panel of Figure 3.

### Results

The mean times spent in the vicinity of the novel and familiar objects by each group in the test phase are shown in the lower panel of Figure 3. A two-way ANOVA of mean exploration times over the four test trials, with group and object as factors, showed significant main effects of object, F(1, 25) = 22.98, p < 0.001. Post hoc pairwise comparisons showed that each group discriminated the novel from the familiar object, ps < 0.05. There was also a main effect of group, F(2, 25) = 14.31, p < 0.001, but no object × lesion interaction, F(2, 25) = 0.24, p = 0.79. For the main effect of group, pairwise comparisons showed that group HPC spent more time exploring objects overall than groups Sham and DLS, ps < 0.001, though groups Sham and DLS did not differ, p > 0.5.

### Discussion

Several rodent studies have shown that the HPC is necessary for SOR in which an animal must integrate object identity with spatial (Aggleton and Nelson, 2020; Bussey et al., 2000; Good et al., 2007; Save et al., 1992), featural (Good et al., 2007; Mumby et al., 2002) or temporal (Good et al., 2007) information. Conversely, numerous studies have demonstrated that rodents with hippocampal lesions are not impaired in standard object recognition (Ainge et al., 2006; Mumby et al., 2002). The results of Experiment 1 add to the body of evidence implicating the HPC in object-location memory, while extending our knowledge of how location is represented. The results of Experiment 2 confirmed that the lesions did not affect standard object recognition memory, while equating some of the procedural and contextual changes encountered by rats in Experiment 1.

As predicted, group Sham discriminated locations with reference to the local geometric context in which an object was first encountered. The result replicates the findings of Poulter et al. (2013), though it should be noted that the current study had a substantially smaller sample size, so statistical power was weaker. One way to ensure that the results in the current study replicated those of Poulter et al. (2013) is to calculate the replication Bayes factor (BF), as described by Ly et al. (2019). The Sham group in Experiment 1 is a direct replication of Poulter et al.’s (2013) Experiment 2. Therefore, taking a single measure of discrimination, the d2 score (novel − familiar/novel + familiar), from both Poulter et al.’s (2013) study and the sham data from the current study, we calculated the replication BF_10_. This was done by calculating the BF_10_ of the combined original and current d2 scores, compared to a chance level of zero. Following Ly et al. (2019), this combined BF_10_, BF_10_ = 17.586, was divided by the BF_10_ of the original d2 scores, also compared to chance, BF_10_ = 5.759. The result, BF_10_ = 3.05, is the replication BF_10_ and indicates that the sham data provide evidence for replication three times greater (precisely, 3.05 times greater) than for the alternative of no replication. We are therefore confident that the sham results reflect a genuine effect.

In terms of recognition memory for object location, the results are important in understanding the cues necessary to define a spatial location in recognition memory. In previous object-location recognition memory procedures, the location of a familiar object is often swapped with that of another or simply displaced (e.g. Dix and Aggleton, 1999; Good et al., 2007), meaning that both the relative positions of the objects, with reference to other objects in the array, and the absolute positions of the objects, with reference to room cues, could be used to define spatial location (see also Langston and Wood, 2010; Wilson et al., 2013, for evidence that egocentric strategies may underlie some object-location memory). Our results demonstrate that the local geometric context in which the object was encountered is sufficient for object-location recognition memory since both the absolute and relative positions of the objects changed between the exploration and test phases. It should be noted that we did not record exploration of objects during the sample phase. It might be argued that differential group exploration during the sample phase might have caused differences in the results that are not due purely to the effects of the lesions on the representation of geometry. However, had the lesions produced a systematic change to the way the lesion groups explored objects, we would have expected a difference between the groups in Experiment 2 as well, which served as a control condition. In addition, a number of other studies (e.g. Ennaceur et al., 1997; Mumby et al., 2002) suggest that lesions to the HPC do not affect exploration of objects in the sample phase of a SOR task. Also, Gaskin et al. (2010) show that sample exploration does not predict test phase performance in SOR tasks.

One of the reasons for conducting our study was the conflicting evidence over the role of the HPC in representing environmental geometry. While experiments with rats seemed to indicate that the HPC was necessary for representing local geometric features, such as the configuration of long and short walls in a particular corner (e.g. Jones et al., 2007; McGregor et al., 2004; Pearce et al., 2004), recent research in pigeons (Sotelo et al., 2019) casts doubt on this conclusion because pigeons showed no transfer between environments based on local geometry, but hippocampal c-Fos analysis indicated hippocampal activity when pigeons experienced transfer to a familiar overall shape. Importantly, the SOR procedure used in our experiments removes the confound of the nature of the motivational demands of the procedure since the pigeon study used an appetitive procedure, while the rat studies used an aversive motivation. The SOR procedure also removes the possibility that the previously reported effects of HPC lesions on learning based on local geometry were because of disruption to the formation of stimulus-response habits in the swimming pool, which were reported by Jones et al. (2007). Our finding that lesions to the HPC disrupted the use of local geometric context for object location in an untrained, nonaversive procedure provides renewed evidence for the role of the HPC in learning based on local geometry (Pearce et al., 2004). It also corresponds with recent evidence from the electrophysiological literature that changing environmental geometry alters the local firing patterns of entorhinal grid cells, which are part of the hippocampal cognitive mapping system, but that more distant grid fields are unaffected by changes to environmental geometry (Krupic et al., 2018).

However, our results also raise the question of why Sotelo et al. (2019) were unable to replicate Pearce et al.’s (2004) findings, but still found c-Fos activation in the HPC. In terms of the use of local geometry, the use of an appetitive task may have had an effect. Golob and Taube (2002) reported rats relying more on a non-geometric landmark than on environmental geometry when motivated by escape from water than when motivated by a food reinforcer: Cheng’s (1986) original finding that rats preferentially relied on geometry over non-geometric features was also based on an appetitive task (but see Lee et al., 2020 for evidence that rats in an appetitive reorientation task also coded the non-geometric features of the environment). The differential effect of motivation has been also observed in the use of spatial strategies: Asem and Holland (2013) showed that rats in a water-submerged plus-maze relied on an egocentric response strategy early in training, switching to an allocentric place strategy later, but found the opposite pattern of results when the maze was drained and the escape platform replaced by the opportunity to find food. Turning to Sotelo et al.’s (2019) report of hippocampal c-Fos activation when pigeons encountered transfer from a rectangle to another rectangle, but no activation when they transferred from a rectangle to a trapezoid, we are only in a position to speculate a reason. One argument is that our lesion study provides stronger evidence of a causal link between the HPC and object-location memory than the correlational nature of IEG activation (Sotelo et al., 2019), though Bingman et al. (2006) showed that hippocampal lesions impaired pigeons’ reliance on geometric cues. Another possibility is that there was a discrepancy between the sensitivity of the behavioural task and that of the neural activation, meaning it was easier to detect changes to IEG expression than it was for behaviour. The possibility also remains that there is a fundamental difference between species in their local and global representations of space. This possibility seems less likely, however, in light of Tommasi and Polli’s (2004) conclusion that chicks represent local geometric features rather than global geometry. Nevertheless, Sotelo et al. (2020) have recently found that the terrestrial toad, *Rhinella arenarum*, also fails to transfer between a rectangle and kite, and Sotelo et al. (2016) showed that c-Fos activation in the medial pallium of the same species, a putative homologue of the mammalian HPC, increased as a result of exposure to environmental geometry. The possibility of between species differences therefore remains. This possibility is made greater by the finding that humans are able to use both local and global representations of space (e.g. Buckley et al., 2016, 2019a, 2019b; Lew et al., 2014; Sturz et al., 2012, 2018), though it should be noted that the role of the HPC in these representations has not been investigated.

In terms of understanding the nature of the impairments to both the HPC and DLS groups in Experiment 1, it is necessary to consider how normal rats represented local geometry. Jones et al. (2007) showed that lesions to the HPC impaired rats’ ability to use the direct metric information provided by wall length. Our results for the HPC group are consistent with this finding and suggest that sham animals retained their ability to represent the different lengths of walls in the two environments. However, unlike in Jones et al. (2007) our use of an untrained procedure prevented sham animals from developing turning habits that seemed to underlie at least some level of successful performance during training. In Experiment 2, there was no impairment in a standard object recognition task, albeit adapted to control for the context change encountered by rats in Experiment 1. Relevant to the results of Experiment 2, O’Brien et al. (2006) and Piterkin et al. (2008) found that HPC lesions impaired the ability of rats to recognise previously encountered objects when the test context was different from the sample context, which we did not find. In light of these findings, our hypothesis about rats coding the local rather than the global geometric properties of the environment is lent more weight as our HPC rats seemed not to detect the change in context, as would be expected if they only coded the local features of corners in which objects were encountered, which were the same across the exploration and test phases.

Turning to the results for the DLS group, we included this group as a positive control since lesions or inactivation of the DLS have tended to impair the formation of habits in spatial memory (Packard and McGaugh, 1996) or the reliance on visual cues in the environment over locations (Devan and White, 1999). In both of these roles, we expected the DLS lesions to have no effect on object-location memory. However, in each of these cases, DLS lesions are thought to impair egocentric coding (White, 2008). It is possible that sham animals in our study constructed the ‘local geometry’ of the environment by means of an egocentric representation. For example, the position of object A in the kite may have been encoded by remembering that it was to the rat’s left in one right-angled corner, relative to a salient feature, such as the end of a long wall, while object B was remembered to the rat’s right in the right-angled corner, relative to the end of the long wall. At test, the unexpected position of object A to the rat’s right at the end of the long wall would have caused dishabituation and renewed exploration, but only if the rat was capable of such an egocentric representation. A number of possible permutations for this kind of egocentric encoding are possible, but if this is the case then successful object-location memory may depend not only on disambiguating long and short walls, involving the HPC, but also coding the object locations with reference to the positions of long and short walls relative to the rats’ own bodies. To our knowledge, only Korol et al. (2019) have explored the role of the DLS in a SOR procedure. They reported that inactivation of the DLS impaired rats’ memory for the identities of previously encountered objects, but not their locations. However, their object-location procedure involved shifting the positions of two previously encountered objects from 40 cm apart to 10 cm apart in an otherwise featureless plexiglass arena. Because objects in our experiment shifted in absolute and relative positions, only the local geometric context could be used to disambiguate objects, which is considerably different from Korol et al.’s procedure.

While our results with DLS lesions are novel, their interpretation does require some speculation which requires further research. Nevertheless, the results of our experiments provide further evidence that animals represent the local geometric features of their environment, and that this encoding is automatic, being evident from recognition memory. This local representation is impaired by lesions to the HPC, which provides further support for the argument that HPC-dependent cognitive maps of the environment are based on local representations of space.
